# Severe Aplastic Anemia following Parvovirus B19-Associated Acute Hepatitis

**DOI:** 10.1155/2017/1359486

**Published:** 2017-04-20

**Authors:** Masanori Furukawa, Kosuke Kaji, Hiroyuki Masuda, Kuniaki Ozaki, Shohei Asada, Aritoshi Koizumi, Takuya Kubo, Norihisa Nishimura, Yasuhiko Sawada, Kosuke Takeda, Tsuyoshi Mashitani, Masayuki Kubo, Itsuto Amano, Tomoyuki Ootani, Chiho Ohbayashi, Koji Murata, Tatsuichi Ann, Akira Mitoro, Hitoshi Yoshiji

**Affiliations:** ^1^Third Department of Internal Medicine, Nara Medical University, Kashihara, Nara, Japan; ^2^Second Department of Internal Medicine, Nara Medical University, Kashihara, Nara, Japan; ^3^Department of Diagnostic Pathology, Nara Medical University, Kashihara, Nara, Japan; ^4^Division of Gastroenterology, Bell Land General Hospital, Sakai, Osaka, Japan

## Abstract

Human parvovirus (HPV) B19 is linked to a variety of clinical manifestations, such as erythema infectiosum, nonimmune hydrops fetalis, and transient aplastic anemia. Although a few cases have shown HPVB19 infection as a possible causative agent for hepatitis-associated aplastic anemia (HAAA) in immunocompetent patients, most reported cases of HAAA following transient hepatitis did not have delayed remission. Here we report a rare case of severe aplastic anemia following acute hepatitis with prolonged jaundice due to HPVB19 infection in a previously healthy young male. Clinical laboratory examination assessed marked liver injury and jaundice as well as peripheral pancytopenia, and bone marrow biopsy revealed severe hypoplasia and fatty replacement. HPVB19 infection was diagnosed by enzyme immunoassay with high titer of anti-HPVB19 immunoglobulin M antibodies. Immunosuppressive therapy was initiated 2 months after the onset of acute hepatitis when liver injury and jaundice were improved. Cyclosporine provided partial remission after 2 months of medication without bone marrow transplantation. Our case suggests that HPVB19 should be considered as a hepatotropic virus and a cause of acquired aplastic anemia, including HAAA.

## 1. Introduction

Acute hepatitis is mainly caused by hepatitis A–E viruses (HAV–HEV), and it is rarely thought to be caused by infection of other viruses, including herpes simplex virus, Epstein-Barr virus, cytomegalovirus, coxsackievirus, echovirus, adenovirus, rubella virus, GB virus, and TT virus.

Human parvovirus (HPV) B19 is a very common viral agent that presents worldwide without ethnic or geographical boundaries. Infection with HPVB19 is known to cause several clinical manifestations, such as erythema infectiosum (fifth disease), transient aplastic crisis, pure red cell aplasia, nonimmune hydrops fetalis, glomerulopathy, and anemia in end-stage renal disease [1, 2]. In addition to these typical symptoms, HPVB19 is associated with acute hepatitis [3]. Although HPVB19-related hepatitis often shows complete and spontaneous remission, particularly in adults, it sometimes induces fulminant hepatitis complicated with acquired aplastic anemia, the so-called hepatitis-associated aplastic anemia (HAAA) [4–8].

Here we report a rare case of severe aplastic anemia following acute hepatitis with prolonged jaundice due to HPVB19 infection in a previously healthy young male.

## 2. Case Report

A 17-year-old male was admitted to Bell Land General Hospital with a 2-week history of nausea and fatigue. He had neither significant drug history nor past medical history, including liver dysfunction. All vital signs were normal, and his consciousness was not impaired. He appeared to be systemically icteric, but there was no evidence of erythema. Abdominal palpitation revealed hepatomegaly, but splenomegaly was not observed.

**Figure 1:**
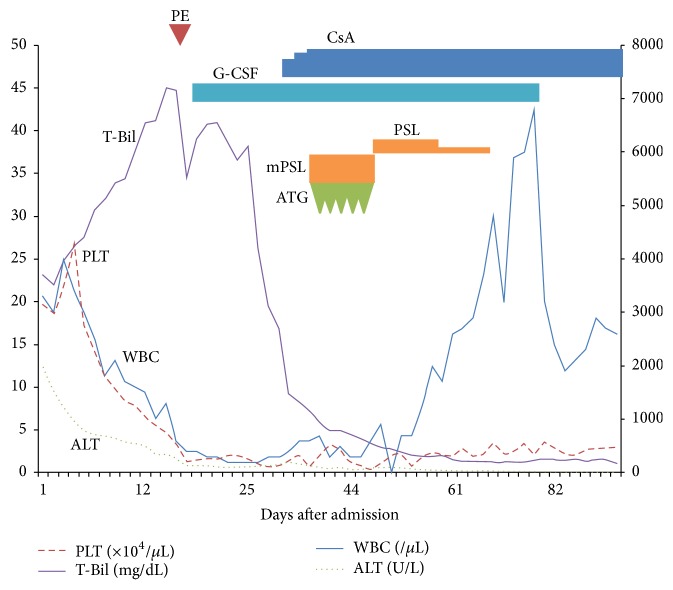
The change in laboratory investigation and the course of treatment. PLT, platelet; T-Bil, total bilirubin; WBC, white blood cell count; ALT, alanine aminotransferase; PE, plasmapheresis; G-CSF, granulocyte colony-stimulating factor; CsA, cyclosporine; ATG, anti-thymocyte globulin; mPSL, methylprednisolone; PSL, prednisolone.

His development after hospital admission is shown in Figure 1. Laboratory investigation on admission (day 1) revealed an extremely elevated aspartate transaminase (AST) level of 2,432 U/L, alanine transaminase (ALT) level of 1,950 U/L, and total bilirubin (T-Bil) level of 23.1 mg/dL. Prothrombin time (PT) activity declined to 30.4% (international normalized ratio, 1.94). Initially, his blood cell count was almost within the normal limits, with white blood cell count (WBC) of 33 × 10^2^/*μ*L, hemoglobin (Hb) level of 14.6 g/dL, and platelet count (PLT) of 19.6 × 10^4^/*μ*L. The serologic test showed negative findings for anti-HAV immunoglobulin M (HAV IgM), HB surface antigen, HB core IgM, HCV IgG, HEV IgA, cytomegalovirus IgM, Epstein-Barr virus IgM, and human immunodeficiency virus IgM/IgG antibodies. Both antinucleic and antimitochondrial antibodies were also negative (Table 1). Ultrasonography demonstrated hepatomegaly without evidence of biliary obstruction, hepatic vein occlusion, ascites, or splenomegaly. During hospitalization, AST and ALT levels gradually decreased, although the decline in PT activity was prolonged and the T-Bil level was markedly increased. Moreover, at day 10, a complete blood count showed WBC of 17 × 10^2^/*μ*L, Hb level of 11.9 g/dL, and PLT of 8.4 × 10^3^/*μ*L, indicating the development of pancytopenia. Because his condition was exacerbated despite plasmapheresis and his pancytopenia was suspected of being myelopathy-derived, he was transferred to Nara Medical University Hospital on day 18.

**Figure 2:**
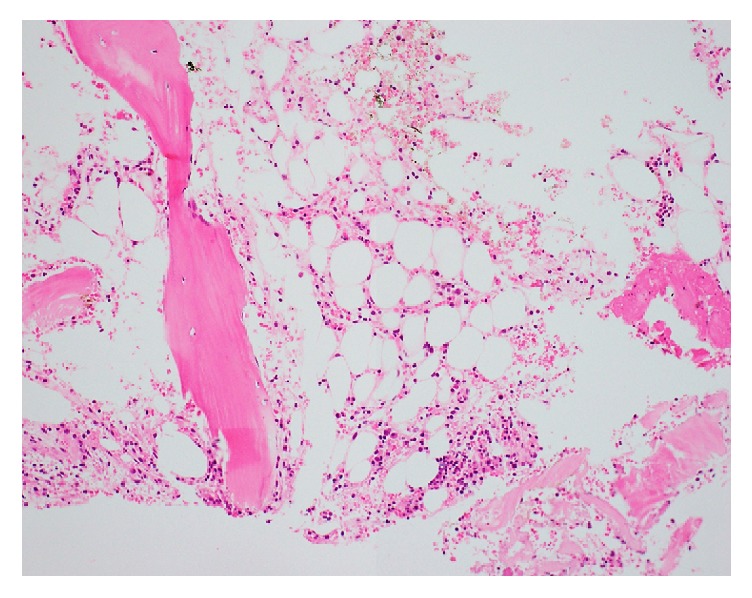
Representative picture of H&E stained bone marrow tissue specimen (original magnification, ×100). His bone marrow showed severe marrow hypocellularity.

Initial laboratory examination after transfer demonstrated an improved AST level of 97 U/L, ALT level of 127 U/L, and PT activity of 62%, whereas an elevated T-Bil level at 34.5 mg/dL was still observed, and pancytopenia grossly progressed to WBC of 4 × 10^2^/*μ*L, Hb level of 9.2 g/dL, reticulocyte count of 4.6 × 10^4^/*μ*L, and PLT of 1.3 × 10^4^/*μ*L. Bone marrow examination showed fatty replacement and hypocellularity, a nucleic cell count of 7000/*μ*L, and no aberrant karyotype (Figure 2). Additional tests for herpes simplex virus IgM, varicella-zoster virus IgM, echovirus type 3 (HI), human T-cell lymphotropic virus 1 IgG, and antineutrophilic cytoplasmic antibodies were all negative. Meanwhile, HPVB19 IgM (EIA) was positive with an optical density value of 3.73 (reference values: <0.8, negative; 0.8–0.99, equivocal; and ≥1.0, positive). HPVB19 DNA was positively detected by quantitative polymerase chain reaction, in agreement with the high titer of HPVB19 IgM antibody (Table 1). Therefore, he was diagnosed with HAAA induced by HPVB19 infection.

When liver injury and jaundice improved with conservative treatment and alimentation, we initiated oral administration of cyclosporine as remission induction therapy for HAAA at 2.5 mg/kg/day on day 32 and gradually increased the dosage to 3.5 mg/kg/day, adjusting trough levels to 150–250 ng/mL. However, there was only slight improvement, and consequently both antithymocyte globulin (ATG) and methylprednisolone were administered in combination with cyclosporine at a dosage of 2.5 mg/kg/day and 2 mg/kg/day, respectively, from day 42 to 46. We continuously administered methylprednisolone until day 70 with gradual tapering of dose, and we treated the patient with granulocyte colony-stimulating factor and transfusion on demand. On day 98 after remission induction with cyclosporine, his pancytopenia improved with WBC of 26 × 10^2^/*μ*L, reticulocyte count of 9.6 × 10^4^/*μ*L, and PLT of 3 × 10^4^/*μ*L without bone marrow transplantation. At present, he is continuously treated with cyclosporine as an outpatient of our hospital.

## 3. Discussion

HPVB19 is the first known human virus in the Parvoviridae family, genus* Erythroparvovirus*, which is a nonenveloped, icosahedral virus containing a single-stranded linear DNA genome [9]. HPVB19 infection rarely presents any symptoms in most immunocompetent individuals, but it causes several well-known clinical manifestations, including erythema infectiosum, arthropathy, transient aplastic crisis, nonimmune hydrops fetalis, meningitis, encephalitis, and myocarditis, particularly in childhood [10]. Symptoms usually begin 6 days after exposure and last for approximately a week. Recently, it has been reported that HPVB19 infection is considered as one of the causes of acute hepatitis [11]. Yoto et al. reported a case of pediatric acute hepatitis in the course of erythema infectiosum, and a case of cryptogenic acute hepatitis without exanthema was suspected to be induced by HPVB19 [12]. Mihály et al. also reported that HPVB19-related hepatitis may occur in 4.1% of patients infected with this virus [13]. Liver damage associated with HPVB19 shows a wide spectrum of disease severity from transient elevation of transaminase levels to fulminant liver failure. However, liver dysfunction induced by HPVB19 is often improved spontaneously in general cases and less frequently leads to a serious condition.

The pathogenic mechanism of hepatic injury by HPVB19 infection has not been elucidated. There are two theories: one is direct viral invasion and the other is an indirect immunological response, namely, virus-associated hemophagocytic syndrome (VAHS) [11]. HPVB19 can infect cells that possess globosides, which are glycosphingolipids acting as the receptor for HPVB19, such as erythroid precursors, megakaryocytes, endothelial cells, and hepatocytes [14, 15]. HPVB19 directly enters the hepatocytes through globosides and produces nonstructural protein (NS1) without the production of viral progeny [16]. NS1 expression significantly upregulates p21/WAF1 expression, a cyclin-dependent kinase inhibitor that induces G1 arrest leading to apoptosis by activation of caspase-3 and caspase-9 [17, 18]. On the other hand, HPVB19 infection reportedly induces VAHS, which increases circulating CD8^+^ cytotoxic T cells and IFN-*γ* and TNF-*α* secretion, triggering symptoms such as high fever, liver injury, enlarged liver and spleen, coagulation factor abnormalities, pancytopenia, and a build-up of histiocytes in various tissues resulting in the destruction of blood-producing cells [19–21]. In the present case, the bone marrow did not show hemophagocytosis but showed aplastic anemia, indicating that VAHS did not primarily participate in the onset of acute hepatitis.

Our patient progressively developed aplastic anemia following severe hepatitis, which is defined as HAAA. This is a well-known and distinct variant of acquired aplastic anemia, in which acute hepatitis leads to marrow failure and pancytopenia [22–24]. HAAA is associated with immunological abnormalities mediated by CD8^+^ Kupffer cells [25]. Patients with HAAA show a decreased ratio of CD4/CD8 cells and a high percentage of CD8^+^ cells, and the residual CD8^+^ cells in the bone marrow produce large amounts of IFN-*γ* [26]. HAAA has been reported in 2%–10% of cases of aplastic anemia [27]. Etiological factors have been attributed to pathogenic viruses, autoimmune responses, liver transplantation, bone marrow transplantation, radiation, and drugs administered to regulate the viral replication, whereas it has been reported that the causal virus was unidentified in majority of cases of HAAA in Japan.

A relationship between HAAA and HPVB19 infection is also controversially described. Langnas et al. have shown that HPVB19 is a possible causative agent of fulminant liver failure and HAAA, while Wong et al. advocated that there is no pathophysiological association [8, 28]. In the present case, it was not definitively concluded that HPVB19 infection was involved in the development of HAAA because we were unable to perform liver biopsy because of the patient's hyperbilirubinemia and thrombocytopenia. If we had the opportunity to perform liver biopsy, we could evaluate the existence of HPVB19 by immunohistochemistry or quantitative polymerase chain reaction.

Clinical guidelines for HPVB19 infection treatment have not been established as most of the symptoms, including liver dysfunction, frequently recover without any treatment. However, HAAA progresses rapidly and is usually fatal if untreated; that is, the mean survival rate of progressed severe bone marrow aplasia is 2 months, and the fatality rate ranges from 78% to 88% [29–31]. Therefore, therapeutic intervention is urgently required for the survival of patients developing HAAA. The primary curative option for treatment of severe HAAA is immunosuppressive therapy [32]. The response rate to immunosuppressive therapy is reportedly 70% [22]. Brown et al. have demonstrated that immunosuppressive therapy with cyclosporine and ATG provides a beneficial outcome in patients with HAAA [27]. Successful treatment with immunosuppressive therapy is usually associated with rapid resolution of acute hepatitis in patients. Cyclosporine and ATG may improve hepatitis as well as bone marrow failure via suppression of cytotoxic T lymphocytes [19]. In addition to immunosuppressive therapy, bone marrow transplantation is also a critical option for the treatment of HAAA. Doney et al. reported 85% survival in patients treated with hematopoietic cell transplantation [32]. Safadi et al. also demonstrated that no cases of recurrent hepatitis occurred during the bone marrow transplantation follow-up period, with patients having reasonable survival rates [33].

In conclusion, HAAA is a distinct clinical syndrome characterized by the onset of bone marrow failure following acute hepatic injury through immunologic mechanisms. The causal trigger of HAAA mostly appears to be an undetermined virus, and, in the present case, HPVB19 is strongly considered as a candidate virus. Most unrecognized, and thus untreated, cases show extremely poor prognosis. Immunosuppressive therapy is reportedly effective, but the long-term outcome for patients with HAAA treated with immunosuppressive therapy is still obscure. Our case suggests that HPVB19 should be considered as a hepatotropic virus and a cause of acquired aplastic anemia. With the accumulation of cases in the future, further elucidation of the disease state and establishment of a treatment method for HPVB19-related hepatitis and HAAA are needed.

## Figures and Tables

**Table 1:** Initial acute hepatitis workup.

Hepatitis A IgM	Nonreactive
Hepatitis B core IgM	Nonreactive
Hepatitis B surface antigen	Nonreactive
HBV-DNA (real-time PCR)	Negative
Hepatitis C IgG antibody	Nonreactive
HIV 1 & 2 antibody	Nonreactive
HTLV 1 & 2 antibody	Nonreactive
CMV antigenemia (C7-HRP)	Negative
HSV IgG antibody (EIA)	Positive
HSV IgM antibody (EIA)	Negative
VZV IgG antibody (EIA)	Positive
VZV IgM antibody (EIA)	Negative
EBV IgG antibody (FA)	Positive
EBV IgM antibody (FA)	Negative
EB nuclear antigen (EBNA) IgG (FA)	Positive
EBV ultraquantitative	Negative
Echo virus type 3 antibody (FA)	Negative
Parvovirus B19 IgM antibody (EIA)	Positive
Parvovirus B19 DNA PCR	Positive
Antinuclear antibodies	Negative
Antimitochondrial antibodies	Negative
TSH (*μ*IU/ml)	0.48
FT3 (pg/ml)	1.5
FT4 (ng/ml)	1.24
PR3-ANCA (U/ml)	Negative
MPO-ANCA (U/ml)	Negative
Ferritin (ng/ml)	1199.5
Iron (*μ*g/dl)	211
TIBC (*μ*g/dl)	227
Ceruloplasmin (mg/dl)	24.4
IgG (mg/dl)	967
IgA (mg/dl)	182.1
IgM (mg/dl)	90.7
AFP tumor marker (ng/ml)	4835.6
HGF (ng/ml)	3.59
